# Reconciling the theory and reality of shared decision‐making: A “matching” approach to practitioner leadership

**DOI:** 10.1111/hex.12853

**Published:** 2018-11-26

**Authors:** Stephen L. Brown, Peter Salmon

**Affiliations:** ^1^ Department of Psychological Sciences University of Liverpool Liverpool UK

**Keywords:** cancer, clinical communication, leadership, patient‐practitioner relationship, shared decision making

## Abstract

Shared decision making (SDM) evolved to resolve tension between patients’ entitlement to make health‐care decisions and practitioners’ responsibility to protect patients’ interests. Implicitly assuming that patients are willing and able to make “good” decisions, SDM proponents suggest that patients and practitioners negotiate decisions. In practice, patients often do not wish to participate in decisions, or cannot make good decisions. Consequently, practitioners sometimes lead decision making, but doing so risks the paternalism that SDM is intended to avoid. We argue that practitioners should take leadership when patients cannot make good decisions, but practitioners will need to know: (a) when good decisions are not being made; and (b) how to intervene appropriately and proportionately when patients cannot make good decisions. Regarding (a), patients rarely make decisions using formal decision logic, but rely on informal propositions about risks and benefits. As propositions are idiographic and their meanings context‐dependent, normative standards of decision quality cannot be imposed. Practitioners must assess decision quality by making subjective and contextualized judgements as to the “reasonableness” of the underlying propositions. Regarding (b), matched to judgements of reasonableness, we describe levels of leadership distinguished according to how directively practitioners act; ranging from prompting patients to question unreasonable propositions or consider new propositions, to directive leadership whereby practitioners recommend options or deny requested procedures. In the context of ideas of relational autonomy, the objective of practitioner leadership is to protect patients’ autonomy by supporting good decision making, taking leadership in patients’ interests only when patients are unwilling or unable to make good decisions.

## 1 INTRODUCTION

Many medical decisions involve choices where outcomes are uncertain. A core aspiration of health policy is that patients should participate in these decisions.^1^ This aspiration expresses the Western cultural commitment to self‐determination as guaranteeing individual autonomy and thereby ensuring that decisions reflect patients’ values and priorities.^2^ There is also an empirical case for patient participation because it can enhance clinical outcomes by generating better clinical information and treatment adherence.^3^, ^4^

Shared decision making (SDM) theory evolved to resolve the tension between patient self‐determination and practitioners’ responsibility to ensure that decisions are evidence‐based and in patients’ interests. SDM approaches based on negotiation between patients and practitioners now dominate the academic literature on how to achieve patient participation. SDM regards patients and practitioners as equal partners, providing complementary insights into the decision.^5^, ^6^ From this perspective, practitioners use their professional expertise to present and explain options to patients; patients use this information to develop preferences based on their values and priorities; and practitioners and patients negotiate a final shared decision. The negotiation model assumes that patients can participate in decision making and that, given accurate and understandable information, they can reach preferences that reflect their values and priorities.^6^ Thus, interventions to promote SDM typically focus on information provision^7^, ^8^ and support for patient deliberation.^9^

Nevertheless, despite over three decades of SDM theory and research, its influence on clinical consultation remains very limited.^1^, ^10^, ^11^ Here, we focus on cancer care as an example of settings where decisions can have profound implications, including survival and quality of life. We argue, in line with Cribb and Entwistle's recent critique of SDM theory and recommendations,^12^, ^13^ that practitioners and patients often cannot collaborate in ways that SDM literature typically prescribes because the assumption that patients are able to make complex decisions about care can be unrealistic. Kleinman^14^ warned that general principles, such as those underpinning SDM, risk becoming merely utopian ideals unless they are firmly based on understanding how the dilemmas they are intended to solve arise, and how practitioners and patients address them in practice. For Kleinman, theory and practice are reciprocally related, with practice providing opportunities to develop and test principles, and those principles providing insights to improve practice. Therefore, in presenting a formulation of SDM that builds on Cribb and Entwistle,^12^ we draw both on empirical accounts of how decisions arise in clinical consultations in cancer care and on contemporary theory in decision making and medical ethics.

## 2 CHALLENGES TO SDM: THE INHERENT ASYMMETRY OF CLINICAL RELATIONSHIPS AND THE INHERENT INFORMALITY OF PATIENTS’ DECISION MAKING

Empirical evidence about how decisions typically arise in consultations in cancer care present two challenges to current conceptualizations of SDM. First, clinical relationships are asymmetric.^15^ Faced with complex and sometimes time‐pressured decisions, patients are often too shocked or distressed to participate, or lack the necessary technical knowledge.^16^ By contrast, they know that their practitioners have the expertise and authority to help them. Thus, patients trust their practitioners to help them feel safe by taking control and making decisions or guiding them.^17^, ^18^ Practitioners can see the clinical relationship in a complementary way, recognizing that patients and their families need to be able to trust them to take control and make decisions.^17^, ^19^ In cancer, therefore, practitioners commonly take leadership roles in decision making by making decisions themselves (often seeking patient assent rather than participation^17^) or recommending options to patients.^13^

Cribb and Entwistle^12^, ^13^ argued that SDM theory and guidance should incorporate the need for practitioners often to lead patients in decision making. Observing that patients’ preferences are sometimes reluctantly provided, ill‐formed or labile, Cribb and Entwistle proposed that practitioners could collaborate with patients to develop well‐considered preferences, rather than simply respond to their expressed preferences. Employing the concept of relational autonomy,^20^, ^21^, ^22^ which describes how selfhood can be nurtured within personal, social and cultural environments that constrain full self‐determination, they argued that patients’ autonomy is supported when practitioners promote well‐considered preferences (see also^23^, ^24^). That is, practitioners should interact with patients supportively, but also critically, to coconstruct preferences. There might also be situations where patients cannot take part in coconstruction, and need practitioners to take responsibility for decisions.^17^ In the context of clinical decision making, therefore, patients’ autonomy might be expressed in feelings of “ownership” and commitment to preferences that practitioners have helped shape, or even to decisions that they have made.^12^, ^13^, ^17^ That is, where patients cannot, or do not wish to, make decisions, their autonomy can lie, not in an explicit role in decision making, but in trusting practitioners to lead decisions.^17^

However, the divergence between clinical reality and the way that SDM has typically been presented should not mean uncritically setting SDM aside and endorsing current practice. First, there are casualties of routine practice in that, just as there are patients who want less involvement than practitioners give them, there are some who need more, for example, because they have knowledge or priorities of which their practitioners are unaware, or because they cannot trust practitioners to make the right decisions.^17^, ^25^ Second, patients’ needs are not homogeneous or constant, even within a single clinical population, and practitioners can find it hard to anticipate these needs or can misunderstand them.^2^ Moreover, many patients’ needs around decision making change over time in ways that practitioners cannot realistically be expected to appreciate.^26^ Finally, relying on practitioners to represent patients’ interests risks biasing decisions to practitioners’ or institutional interests. Unsurprisingly, therefore, many practitioners want guidance on when and how to lead decision making.^13^, ^27^

If we accept that decision making can legitimately take different forms, from full self‐determination to accepting practitioners’ decisions, the challenge is to develop an approach to practice that helps practitioners recognize when and how it is appropriate to lead decision making. Our starting point is the argument^12^, ^13^ that achieving the ethical goals of SDM requires practitioners who are prepared to be more directive than most models of SDM suggest. Our formulation offers the “practical steps” that Cribb and Entwistle^12^ call for to help reconcile the imperative to avoid the dangers of unfettered paternalism with the freedom that practitioners need if they are to provide ethically defensible leadership.

A second challenge to SDM arises from evidence of the informality of patients’ typical decision making, because this is hard to reconcile with the assumption, central to SDM, that normative standards exist for “good” decisions. The quality of a decision cannot be judged from its consequences, which are unknown when the decision is being made, but has to be judged from what is known at the time about decision processes.^30^ Early approaches to assessing quality of decision making processes were based on “likelihood‐value” models, which assumed that people made decisions rationally so that decisions reflected estimation and aggregation of the specific risks and benefits associated with available choices. However, abundant evidence shows that patients think about risk “informally,” that is they use reasoning heuristics—idiographic and context‐dependent cognitive “shortcuts” or simplifications—that reduce complex and demanding cognitive tasks to simpler and more easily understood tasks.^31^, ^32^, ^33^, ^34^, ^35^ Examples include the “affect heuristic,” whereby people infer risk from their emotional responses such as fear or dread,^36^, ^37^ and the “availability heuristic,” whereby risk perception is influenced by how readily information can be recalled.^38^ In over‐simplifying complex information, heuristics can introduce factual biases and logical errors.^39^ Some authors even argue that heuristic reasoning is irrational and therefore threatens patient autonomy.^23^, ^24^

Informal reasoning should not, however, be regarded as aberrant. First, heuristics are not confined to isolated lapses in otherwise logical decision processes, but people routinely use heuristics to encode and process risk information during decision making.^32^ Moreover, heuristics can facilitate high‐quality decision making. In laboratory decision research, decision quality is defined as the extent to which participants’ decisions are consistent with those pre‐defined as being correct through either formal logic or expert opinion. Using these criteria, informal decision making often leads to better decisions.^32^, ^33^, ^34^ The superiority of informal thinking has been attributed to it allowing more selective information uptake and simpler decision making by comparison with formal logic which is often too cognitively demanding for people to implement effectively.^32^, ^33^ Nevertheless, appreciating that patients use informal reasoning creates a formidable problem for SDM. Normative standards for “good” reasoning become elusive because informal thought is idiographic and contextualized.

## 3 TOWARDS A NEW FORMULATION OF SDM: A PRACTICABLE APPROACH FOR PRACTITIONERS TO JUDGE THE QUALITY OF PATIENTS’ INFORMAL DECISION MAKING

Recognizing that patients make decisions based on informal and idiosyncratic reasoning that does not necessarily follow logical rules, Elwyn and Myron‐Schatz^30^ proposed a two‐part definition of a “good” decision process, according to which patients should: (a) recognize that choice exists between mutually exclusive options, and that the outcomes of these options are relevant to them; and (b) form views on the desirability of those outcomes. Elwyn and Miron‐Shatz explicitly were not concerned with *how* patients select, understand and integrate information to arrive at their decision. Therefore, their definition of a good decision sets up a “black box” problem because patients might satisfy the definition by recognizing that options are available and by developing views on the desirability of their different outcomes, but those views might be distorted by incorrect beliefs or logical inconsistencies.

Assessment of decision quality therefore arguably needs to go further than Elwyn and Miron‐Shatz’ criteria, to include attention to the content of patients’ reasoning—that is, the “workings” of the “black box.” Recognizing the importance of heuristics in patients’ reasoning offers an approach to “opening” the black box. Heuristic thinking generates mental representations of risk‐related information that permit decision making,^35^ and those representations can be elicited and assessed, as fuzzy‐trace theory (FTT^35^, ^36^) explains. FTT uses the term “gists” to describe these mental representations. Formed after exposure to risk‐relevant information, gists are simplified representations of risk that encapsulate the significance of that information. For example, when given a numeric breast cancer risk estimate, a woman may encode this, not as a numerical probability, but as a feeling of being at “high risk,” “vulnerable” or “greater risk than average”.^37^ As simplified representations of risk, such gists allow rapid decision making. FTT further proposes that risk‐related gists take the form of idiographic propositions concerning the likelihood of outcomes and their value.^38^, ^39^ Several researchers have shown that these propositions predict people's behaviour in relation to risks,^38^, ^39^, ^40^ and that people routinely use gists to make medical 41 and non‐medical^34^ decisions. FTT uses the term proposition to signify “hypotheses,” or *prima facie* beliefs about risk, that are modifiable by evidence or counterargument.^38^ Recognizing the importance of propositions provides a way for practitioners to open the “black box” of informal decision making. They can elicit and evaluate the propositions that patients are using to make decisions, for example, by asking questions such as “Could you tell me about the reasons you decided this?” or “What did you think about when making this decision?”

Taking Elwyn and Myron Schatz's^30^ first criterion for “good” decision making, that patients understand options and their relevance to the clinical problem, we can add therefore that the propositions they are drawing on should be *reasonable*. Propositions about risk, such as “I have a real chance of cancer,” are not numeric and thus do not correspond to objective risk (when it is known) in the way that probability estimates do. Thus, reasonableness must be a clinical judgement that takes account of the nature of the decision and its context. For example, breast cancer patients deciding between surgical procedures could hold the simple proposition that “mastectomy is more likely than lumpectomy to save my life because it removes more tissue”.^42^ Having elicited this proposition, the practitioner can evaluate it: Will mastectomy improve this patient's life expectancy? Where it is false, the practitioner can challenge it, lest it leads a patient to choose more invasive surgery for no additional benefit.

Elwyn and Myron Schatz's^30^ second criterion for a good decision is that patients form views on outcome desirability. The value that patients put on outcomes is, of course, anchored in their own priorities which cannot be checked against external criteria. Therefore, the practitioners’ task is to judge whether value‐related propositions are reasonable given patients’ own values and priorities. For example, when women at risk of breast cancer decide about risk‐reducing mastectomy, some are motivated by believing that “I must do everything I can to prevent cancer recurring” or that “only the most drastic action will reassure me”.^43^ Practitioners might think these propositions unreasonable if they prevent women considering the risks of surgery or the possibility that non‐surgical options could mitigate risk.

A propositional view therefore extends Elwyn and Miron‐Shatz's^30^ proposal on judging decision quality because it addresses the problem of whether patients’ views are reasonable or not. Argumentation theory^44^ provides an ethical and empirical approach to eliciting, understanding and attempting to modify patients’ (and practitioners’) propositions. The theory describes the ethical responsibility for practitioners and patients to provide clear justifications for their preferences, and to elicit and take account of, but also to point to weaknesses in, the other party's views. This approach is supported by evidence that patients in primary care or clinical trials research can, indeed, provide arguments if asked, and that they are more likely to consider the views of practitioners who, correspondingly, provide evidence‐based and logical arguments for their positions.^45^, ^46^ In argumentation, reasonableness is based on understanding the person and context.^44^ It is not normative and cannot be objectively defined—practitioners must make the best judgements they can, given the evidence. Nevertheless, we see practitioners’ subjective judgements of reasonableness as the key to identifying a “good” patient decision. This approach to judging decision quality provides a platform from which we can return to the problem of when and how practitioners should lead decisions.

## 4 A “MATCHED LEADERSHIP APPROACH” TO SDM

Equipped with the theoretically based approach, described in the previous section, that practitioners can take to judge the quality of patients’ decisions, we can now address the challenge of fashioning guidance for practitioners as to when and how they should lead decisions that maximize patients’ autonomy while protecting their interests. As the basis for this guidance, we return to the concept of “relational autonomy,” which recognizes that patients’ choices are inherently limited by their context, and that autonomy lies, not necessarily in the frequently unrealistic ethic of self‐determination, but in the interpersonal relationships, particularly with practitioners, which support patients and enable them to make decisions.^22^, ^28^ A relational autonomy perspective also emphasizes that patients who can make independent decisions ought to be afforded that opportunity.^29^ We propose three principles as foundations of guidance about how practitioners can gauge leadership so as to maximize patients’ autonomy.

### 4.1 It is sometimes right for practitioners to lead decision making

This position needs to be stated, and widely acknowledged, because the priority currently given to self‐determination risks promoting a culture that eschews practitioner leadership. When, as Cribb and Entwistle^12^ argue, patients cannot or will not form preferences and make decisions reasonably, or want practitioners to help them, practitioners’ leadership of decision making can protect patients’ interests. Further, practitioners should not be tentative about taking leadership if they believe that patients’ decisions are unreasonable. Sometimes, this may lead to disagreement with patients. Of course, practitioners should try to reach agreement, but they will ultimately need to act on their own views of what is reasonable for any specific patient, to protect patients’ interests where their views diverge from patients’.^47^

### 4.2 Leadership should be proportionate and aim to maximize patient participation

Patients are better placed than practitioners to know their own priorities.^2^, ^48^ Where views diverge, a potential cost of practitioner leadership is that the focus of decision making moves from patients’ values and goals to those to that practitioners, sometimes wrongly,^2^ ascribe to them. Therefore, practitioners’ assumption of leadership needs to be proportionate—minimizing their leadership to allow patients to steer decisions as much as possible and, when practitioners do assume leadership, ensuring that they are aware of patients’ values and take these into account.

### 4.3 Leadership should develop each patient's future capacity to make decisions

Several commentators have taken a relational autonomy perspective to argue that practitioners have a responsibility to help patients develop their own decision making capacity 28, 29 but there is little explicit guidance as to how they can do so. Patients with cancer or other serious illness can face multiple and inter‐related decisions over the trajectory of their illness, so any instance of decision making provides the opportunity to shape patients’ capacity or motivation for more active roles in future. Specifically, we argue that eliciting and, where appropriate, modifying patients’ propositions could foster more realistic understanding that can better equip patients to contribute to decisions in the future.

## 5 A MATCHED LEADERSHIP APPROACH IN PRACTICE

In our formulation of decision leadership, practitioners’ key role is to ascertain how patients have arrived at their preferences, identifying, evaluating and, where appropriate, questioning or seeking to modify the underpinning propositions. The approach is therefore dynamic, in that the level of leadership a practitioner takes might change fluidly from moment to moment in a consultation as new information about the patient's propositions and decision making arises. The different “levels” of leadership we distinguish below do not denote a hierarchy of superiority, but different degrees of directiveness that a practitioner might take. In contrast with previous approaches to SDM that attempt to prescribe a “correct” model of practice,^5^, ^6^, ^10^ our approach emphasizes practitioners’ thoughtful interpretation of the reasonableness of patients’ views and their flexible and sensitive responses to the information that patients provide. We therefore describe a hierarchy of levels of leadership, with more directive leadership contingent on the inability of less directive leadership to deliver decisions that practitioners judge reasonable. Boxes 1 and 2 illustrate these levels and contrast them with the approach based only on Elwyn and Miron‐Shatz.^30^ We do not assume that no practitioner yet provides leadership in the way we propose. Indeed, we recognize, as have previous advocates of broadening SDM models to include practitioner leadership,^13^ the sophistication of many practitioners in already identifying and taking the lead that their patients need. Therefore, our proposal is intended to help bridge the gap between formal SDM theory and clinically realistic best practice, so that quality decision making can be recognized where it exists—and facilitated where it does not.

Box 1 **Matched leadership approaches to a breast cancer (BC) survivor's request for contralateral risk‐reducing mastectomy (CRRM)**

1

- Dianne underwent mastectomy and chemotherapy to treat cancer in her left breast. She has no genetic mutation that would increase her risk of BC, but her BC history puts her at greater risk of cancer in the right breast than non‐affected women. Dianne requests CRRM.
- CRRM can reduce BC risk but probably will not extend Dianne's life because it cannot prevent metastatic disease. It has surgical and cosmetic risks, and women are sometimes dissatisfied with the outcome, so surgeons are careful in their decisions about whether to provide it.
- According to current SDM theory,^30^ a “good” decision involves Dianne being informed of the range of risk‐reducing options, including CRRM, understanding the potential consequences of choosing CRRM or a different option, and considering how each consequence would affect her. Consistent with this guidance, her surgeon explained surgical risks and presented alternatives to CRRM including hormonal treatments, and their consequences, and explained that CRRM does not prevent metastatic disease. The surgeon asked Dianne to imagine potential consequences of having or not having CRRM, and how she would respond. Dianne still wanted CRRM and the surgeon agreed.
- Although fulfilling her responsibilities according to current SDM theory, the surgeon did not ask Dianne to explain her reasoning, or try to elicit the propositions underlying it. The decision might have unfolded differently had the surgeon followed our recommendations. Consider two scenarios.

**Where propositions are reasonable: “presenting options and informing decisions”**

- When asked to explain the reasons for her decision, Dianne described being highly fearful of further BC. Her surgeon elicited two propositions. CRRM would mean that, even if further cancer arose, “I'd know that I'd done all I could,” and in the event of surgical complications or a poor cosmetic outcome “that couldn't be worse than the feeling I have now, that I can't move on with my life.” She knew the risks of surgery, but felt that non‐surgical options would not help her feel “safe.”
- In talking with the surgeon, Dianne acknowledged that her wish for CRRM was not justified by objective risk reduction, but by her need to control her own fear.
- The surgeon knew that CRRM can reduce fears of cancer recurrence [43], and that there is little evidence that non‐surgical options can. Thus, believing that her propositions were reasonable in the circumstances, the surgeon agreed to CRRM.

**Where propositions are unreasonable: “helping to develop reasonable propositions” and “directive leadership”**

- In this scenario, the surgeon elicited propositions that “I'm destined to get cancer in my other breast,” and that “I couldn't cope with another cancer.” Exploring these identified Dianne's belief that BC was inevitable without CRRM because “it's happened once, so it's lurking in my breast and will creep out again,” and that she was “just too fragile to go through cancer again.” She expected to be free of worry after RRM.
- The surgeon considered these propositions unreasonable. Contralateral BC is not inevitable and non‐surgical options can reduce risk. Evidence suggests that RRM can reduce worry about BC, but does not eliminate it. To *help develop reasonable propositions*, the surgeon showed Dianne evidence that risk reduction by CRRM is small and that it cannot reduce metastatic disease risk. Dianne shrugged off the evidence: “If anyone gets BC, it will be me.”
- The surgeon did not think that Dianne's request for CRRM was based on reasonable propositions, and did not think that she could be helped further to form more reasonable ones. Therefore, taking a *directive leadership* approach, the surgeon declined her request, explaining that this was because CRRM would not give her the benefits she sought and that other approaches would give her the same protection against future cancer as CRRM, while avoiding the risks of surgery.

Examples of propositions taken from Brown et al^40^ and Heineger et al.^54^

Box 2 **Matched leadership approaches to helping a patient to decide about consent for prognostic testing in uveal melanoma**

1

- Uveal melanoma (UM) is a treatable cancer of the eye. It carries a 40‐50% probability of metastasis, which is usually fatal. A prognostic test provides accurate life expectancy estimates over 10 years. Clinical utility of prognostication is limited because contingent screening and treatment for those at high risk do not currently improve life expectancy. Prognostic testing is offered in some clinics because there is little evidence that it causes psychological harm and most patients undergoing it say that they would want it if they had the choice again.^55^ The decision to have or not to have testing is therefore a matter of patient preference. However, it is made under time pressure because it requires a biopsy during cancer surgery, and so must be made before surgery.
- Dennis has been diagnosed with UM and offered the test, which he has accepted. Surgery was planned for two days following diagnosis, so he had to decide quickly. Consistent with current SDM guidance,^30^ the surgeon checked that Dennis understood the potential consequences of having or not having the test, particularly that contingent screening and treatments would not improve life expectancy, and had considered how each would affect him. The surgeon explained options and described possible negative consequences of testing, including emotional distress if the prognosis was poor.
- Although fulfilling responsibilities according to current SDM theory, the surgeon neither asked Dennis to explain his reasoning, nor tried to elicit the propositions underlying it. The decision might therefore have unfolded differently had the surgeon followed our recommendations. Consider two scenarios.

**Where propositions are reasonable: “presenting options and informing decisions”**

- The surgeon elicited two propositions that Dennis used to make important decisions in his life, including this one: “it's better to know than not to know” and “I prefer to face up to reality.”
- Thinking these reasonable and in accord with Dennis’ values, the surgeon accepted Dennis’ consent to the test, having judged that it was unnecessary to go beyond “*presenting options.”*

**Where propositions were unreasonable: “helping to develop reasonable propositions”**

- The surgeon elicited Dennis’ proposition that “there must be a good reason for the test, otherwise it wouldn't be offered.” When prompted further, Dennis assumed that a poor result would lead to potentially life‐saving treatment.
- The surgeon regarded these propositions as unreasonable. *To “help develop reasonable propositions,”* the surgeon reiterated that current treatments for uveal melanoma metastases are not yet effective in prolonging life, and explained that they are offered, instead, only to improve the quality of remaining life.
- After reflecting on this, Dennis concluded that the risk of learning of a poor prognosis that could not be mitigated outweighed the possibility of relief following a good prognosis. He declined the test.

Examples of propositions taken from Cook et al^56^ and Hope‐Stone et al.^57^

*Presenting options and informing decisions* is the first level, appropriate when patients are willing and able to make decisions based on propositions that the practitioner considers reasonable. Practitioners present options, confirm that patients understand these and their relevance to the clinical problem^31^ and, crucially, elicit patients’ propositions to ensure that these are reasonable. At this and subsequent levels, practitioners might suggest an option that they favour, giving reasons, while explaining that the decision is the patient's.^49^

*Helping to develop reasonable propositions* is the level at which practitioners help patients engage with the decision process because they believe that patients’ propositions are unreasonable. The defining feature of this level is that practitioners provide arguments with the aim of helping patients to develop reasonable propositions, but do not directly question or direct the decision by arguing for a specific option. Practitioners might correct inaccurate propositions, suggest options or outcomes that patients have not considered, or prompt patients to reflect on their propositions or the ways in which they have integrated them. This level of leadership would become superfluous once patients either change propositions to ones that the practitioner judges reasonable or when practitioners conclude that they cannot modify unreasonable propositions.

*Directive leadership* is where practitioners shape or make the decision because they judge that patients are drawing on unreasonable and unmodifiable propositions or are unable or unwilling to decide. Practitioners might argue for specific options or seek to exclude options they judge harmful. In this role, practitioners try to integrate what they know of patients’ values and goals with what the evidence suggests about outcomes of different options.^47^ This leadership level differs crucially from the others. In both “*presenting options”* and “*helping patients develop reasonable propositions,”* practitioners trust a patient to make choices that their practitioner would not necessarily have chosen, because the practitioner judges the propositions underlying those choices to be reasonable for the patient. *Directive leadership* entails an obvious risk of “paternalism,” whereby practitioners override patients’ own values and goals. To reduce that risk, practitioners can explicitly elicit patients’ views about their recommendations and modify those recommendations where necessary.^17^ Further, practitioners can explain the reasons for their recommendations and invite patients to review them. As well as providing the opportunity for changing the decision, practitioners’ explanations might thereby develop patients’ capacity to participate in future decisions.

## 6 DISCUSSION AND IMPLICATIONS

Our approach differs from much of the existing literature on SDM. Driven by a self‐determination ethic, this literature emphasizes patients’ participation in decision making above other considerations. By contrast, our approach takes, as the starting point, the tension between respect for self‐determination and a comparable respect for the reality of what it means to be a patient: making decisions informally and needing practitioners sometimes to lead decisions. Our formulation adds to Cribb and Entwistle's^12^ critique by providing a practical approach whereby practitioners can balance cultural expectations about how clinical interactions “should be” with the reality of how interactions “are.”

### 6.1 Implications for practice and research

As Entwistle et al^13^ warned, an approach to SDM that relies upon practitioners’ judgements of when and how to lead decisions makes external assessment of their performance difficult. In our formulation, the roles appropriate for the patient and practitioner in any decision depend on the practitioner's judgement of the reasonableness of the propositions underpinning the patient's choices. This judgement will depend on the specific clinical and social context. It follows that practitioners’ judgements of reasonableness cannot be pre‐specified or assessed against generic criteria. Nevertheless, ethical practice requires that practitioners’ judgements should be defensible as good ones. This dilemma—how to ensure that practitioners can be accountable for judgements that are necessarily partly subjective—is a familiar one in clinical practice broadly. Whereas general guidance and protocols can inform practitioners’ clinical decisions, they cannot always dictate their decisions in individual cases. Decisions about individual patients often need good judgements, particularly where there is uncertainty. Clinical professions have therefore developed ways to foster good judgements by exposing these to scrutiny of peers and by making practitioners accountable for how they reach their judgements. Mental health professions emphasize peer supervision, nursing has advocated reflective practice, and medicine has the arena of the ward round. Moreover, across health care, multidisciplinary team meetings allow practitioners to scrutinize and contest judgements about individual patients. Recent pointers^50^, ^51^ to how peer supervision and scrutiny amongst groups of practitioners can facilitate and inform their reflexivity around clinical communication in cancer care suggest ways in which judgements about leadership might also be opened to reflection, challenge and support. In taking such approaches, the guarantee of ethical practice lies, not just in adherence to external standards, but in the process whereby judgements are scrutinized and contested.^14^

The need to develop practitioners’ reflexivity around decision making demands new thinking about clinical guidance and training. Guidance cannot be prescriptive, but would instead seek to render the framework outlined in this article accessible to practitioners as a way in which they can structure their thinking and reflection about decision making. While a place exists for formal training, including presentations and workshops, to teach the elements of our approach, learning to use it to be reflexive needs to be based in practice, not the classroom. For instance, Salander^51^ described a “Balint” group for cancer practitioners, in which a communication expert facilitated practitioners’ reflections on dilemmas regarding clinical interactions and relationships. Our formulation offers a framework that could structure reflexivity about leadership in decision making in such a setting. Thus, we offer decision making “experts” new opportunities to facilitate and inform practitioners’ reflexivity around their decision making.^51^

Evaluating our proposal invites a broad approach. Many researchers have sought to show that SDM improves patient outcomes such as satisfaction and participation and reduces decisional conflict.^4^ Investigating the effects of our approach on patient outcomes will be important, but outcomes need to be carefully interpreted according to how they reflect our aim—that patients make “good” decisions. For instance, patient participation and satisfaction are commonly studied as desirable outcomes in SDM research, but their value will depend on the context; they might not be achievable or, if propositions are unreasonable, they might even be inimical to good decisions. Clinical outcomes, while also clearly important, do not necessarily indicate decision quality; for instance, patients can make good decisions that lead to poorer clinical outcomes because their decisions were influenced by personal factors, such as differing values they put on duration or quality of life.

Outcome evaluation reflects a consequentialist ethical approach, whereby practitioner behaviours are considered desirable if they produce desirable outcomes. By contrast, Duggan et al^52^ point to the importance of the deontological tradition in bioethics, whereby practitioners’ behaviour is justified as desirable when it is intrinsically “right.” From this perspective, our approach can be evaluated, not only by its outcomes, but according to the validity of its assumptions and arguments in characterizing what patients need from their practitioners. Central to the validity of our argument is the role of patients’ propositions in decision making. Therefore, an important implication of our approach is that more needs to be understood about the propositions that patients hold when making specific decisions,^39^, ^40^, ^41^ and the extent to which practitioners can elicit these (or be helped to elicit them). Practitioners’ judgements of the reasonableness of patients’ propositions also need investigation to identify how practitioners approach such judgements and to what extent different practitioners can converge on common judgements. There is a developmental element to our approach that also needs investigation. We argue that practitioners’ leadership stance in any instance should enhance patients’ future decision making capacity. Longitudinal research can test this and explore how the development of capacity can best be facilitated.

However, our approach demands more of research than just a focus on patients’ propositions. For areas of clinical practice that need to be both empirically informed and ethically justified, “top‐down,” deductive research needs to be balanced by inductive “bottom‐up” research; that is, by careful analysis of the ways in which practitioners and patients navigate the demands, constraints and opportunities of specific settings.^14^, ^53^ Such research exposes practices to researchers, theorists, practitioners and educators, who can examine and critique them from different theoretical and ethical perspectives. This critique will sometimes identify practices that are questionable theoretically or ethically. Correspondingly, both practice and theory will be strengthened where research identifies innovative solutions that practitioners have developed to solve clinical problems in complex and contested areas such as decision making.^17^ Inductive research also helps ensure that practitioners are accountable for their decision making judgements, not just to local peers, but to broader professional and societal interests. For instance, because our approach rests on practitioners having the responsibility to judge when to lead decision making, it might be criticized ethically as consolidating the dangers of unfettered practitioner authority. Exposing judgements to other practitioners in individual supervision or team meetings cannot, alone, counter that criticism, because issues around patient autonomy cannot be owned by any clinical profession, but must reflect broader cultural expectations as well as being informed by available evidence. Thus, the broad range of communication researchers, including social scientists and ethicists, as well as practitioners, has a crucial role in exposing and critiquing practitioners’ strategies in decision making in routine care.

## 7 CONCLUSION

Our approach contributes to an area of ethical debate that current SDM literature often disregards. Many papers continue to lament practitioners’ or patients’ failure to engage in SDM. However, few use this failure as a starting point to question the concept of SDM. Instead, they pursue ways to train, educate or persuade patients or practitioners to perform SDM.^10^ That is, they base their work exclusively on the ethical principle of autonomous self‐determination, although rarely making this explicit. By contrast, by making the opposing ethical priorities that underpin our own approach explicit, we invite the continuing ethical debate that is essential if the concept and practice of SDM is to be clinically realistic as well as ethically robust.
